# Fossil biocalcite remains open to isotopic exchange with seawater for tens of millions of years

**DOI:** 10.1038/s41598-024-75588-7

**Published:** 2024-10-22

**Authors:** Deyanira Cisneros-Lazaro, Arthur Adams, Jarosław Stolarski, Sylvain Bernard, Damien Daval, Alain Baronnet, Olivier Grauby, Lukas P. Baumgartner, Torsten Vennemann, Jo Moore, Claudia Baumgartner, Cristina Martin Olmos, Stéphane Escrig, Anders Meibom

**Affiliations:** 1 Laboratory for Biological Geochemistry, School of Architecture, Civil and Environmental Engineering, Ecole Polytechnique Fédérale de Lausanne, 1015 Lausanne, Switzerland; 2 Institute of Paleobiology, Polish Academy of Sciences, 00818 Warsaw, Poland; 3 Institut de Minéralogie, de Physique des Matériaux et de Cosmochimie, Muséum National d’Histoire Naturelle, CNRS, Sorbonne Université, 75005 Paris, France; 4 Institut des Sciences de la Terre, CNRS- Université Grenoble Alpes, 38058 Grenoble, France; 5 Centre Interdisciplinaire de Nanosciences de Marseille, CNRS - Aix-Marseille Université, 13288 Marseille, France; 6 Center for Advanced Surface Analysis, Institute of Earth Science, University of Lausanne, 1015 Lausanne, Switzerland; 7 Institute of Earth Surface Dynamics, University of Lausanne, 1015 Lausanne, Switzerland

**Keywords:** Palaeoclimate, Palaeoceanography

## Abstract

Fossilized remains of marine calcifiers constitute the physical basis for reconstructions of both deep ocean and sea-surface temperatures going back millions of years, but paleoclimate records derived from their isotope and trace-element chemistry can be biased by diagenesis. Experiments simulating diagenesis in the presence of an ^18^O-rich seawater analogue were conducted with modern and 14 Myr old foraminifera (*Ammonia* sp.) tests to investigate their relative susceptibility to oxygen isotope exchange. The fossilized tests were of exceptional preservation and similar to modern tests in terms of structure and crystalline organization, but had experienced partial loss of embedded organic structures, thus a priori offering fewer preferential pathways for porewaters to penetrate the tests. NanoSIMS imaging revealed that oxygen isotope exchange was pervasive in fossil tests, with isotopic exchange occurring at approximately half the rate of modern tests. The results unequivocally show that fossil biocalcites are metastable and remain more susceptible to isotope exchange than abiotic calcites millions of years after sedimentation and burial.

**Supplementary Information:**

The online version contains supplementary material available at 10.1038/s41598-024-75588-7.

## Introduction

The fossil carbonate skeletons of marine calcifiers, such as foraminifera, bivalves and brachiopods, are essential archives for understanding the evolution of marine environments, and hence global changes in climate over geological time^1–4^. One of several elemental and isotopic proxies, the oxygen isotope paleothermometer^1,5^, has permitted reconstruction of deep-water and sea-surface temperatures from the beginning of the Phanerozoic to the present^6^. Beyond the challenges associated with estimating the isotopic composition of paleoseawater and correcting for ‘vital effects’, accurately reconstructing past ocean temperatures is predicated on the assumption that there has been minimal isotopic exchange between the fossil biogenic carbonates and the surrounding porewaters during and after sedimentation.

The potential for diagenesis to affect the preservation of elemental or isotopic compositions of biogenic carbonates was recognized already in the infancy of paleoclimate reconstructions^1^, with most subsequent work focusing on physico-chemical alterations. In macrofossil research, the screening for diagenesis typically involves assessment of textural and crystallographic changes with optical and electron microscopy, and chemical screening techniques such as changes in luminescence (brought on by diagenetic incorporation of manganese) and alteration of trace element and stable isotope values (see^7^ for a review). Studies of the diagenesis of microfossils such as foraminifera tests have mostly concentrated on structural modifications brought on by dissolution, overgrowth and secondary precipitation^8^. The glassy-frosty paradigm established that fossil tests that appear ‘frosty’ under optical microscopy generally yield strongly biased δ^18^O-based seawater temperature reconstructions due to secondary carbonate precipitation, in contrast to ‘glassy’ tests that are typically derived from hemipelagic clay-rich sites and have preserved a translucent appearance^9,10^. However, Bernard and colleagues^11^ recognized that significant modification of the isotopic composition of foraminifera tests can take place without observable (by optical or scanning electron microscopy) ultrastructural changes to foraminifera tests. Subsequent experiments designed to quantify the rates of biogenic calcite-fluid exchange showed that a ^45^Ca radiotracer can be rapidly incorporated into foraminifera test calcite^12^ and that oxygen isotope compositions of foraminifera tests can be significantly altered through (rapid) grain-boundary diffusion^13^, all without any accompanying ultrastructural changes. In addition, it was demonstrated that calcium, carbon and oxygen isotope exchange between nano- to micrometer sized abiotic calcite crystals and fluid occur at ambient temperatures without any change in grain size^14,15^. These studies indicate that low-temperature diagenesis can change the original isotope and chemical composition of biocalcites, even on geologically short time scales, thus potentially biasing paleoenvironmental reconstructions without leaving any noticeable evidence of this alteration.

In a previous study^16^, diagenesis was simulated in experiments with ^18^O-labelled artificial seawater combined with NanoSIMS high spatial resolution isotopic imaging to understand how fluids interact with calcitic tests of modern hyaline rotaliid foraminifera, the order most commonly used in paleoclimate reconstructions^13^. Pore fluids were found to pervasively penetrate the entire test via organic-rich conduits (common to all rotaliid foraminifera) that act as preferential pathways: cogwheel interfaces and organic linings. The ‘cogwheel’ interfaces, visible on the surfaces of test walls of both modern and fossil foraminifera, separate 3-dimensional domains of distinct crystallographic orientation^17,18^. Organic linings, architecturally similar in all rotaliid foraminifera tests, are layers that serve as templates for calcification. During growth, foraminifera extrude an organic template outlining the new chamber and covering the rest of the outer surface of the test^19,20^. The walls of previously formed – and hence overgrown – chambers thus contain a number of organic linings corresponding to their position in the test. Both cogwheel interfaces and organic linings act as penetration ‘highways’ that bring pore fluids close to all internal parts of the tests, facilitating isotope exchange with the entirety of the test structure, the bulk of which is composed of 10–100 nm sub-spherical calcite particles coated by organic materials^16^. Such nano-scale granular texture is typical of most biominerals from disparate taxa and phyla and is thought to result from a common biomineralization process involving an amorphous calcium carbonate precursor ^21^. Given that 95% of the initial stock of proteins can be degraded in less than 120 kyr^22^, the organic linings in fossil biocalcitic structures can generally be regarded as (at least partially) degraded. Therefore, it could be surmised that fossil biocalcites could be less susceptible to diagenetic isotope exchange than modern counterparts due to the loss of organic-rich conduits for fluid penetration. To investigate this hypothesis, we incubated 14 Myr old *Ammonia* tests^23,24^ for 6 days in ^18^O-enriched seawater analogue at calcite saturation and 90 °C to establish the degree to which fluids can penetrate into and isotopically exchange with fossil tests with partially degraded organic matter. Modern, pristine *Ammonia* tests were incubated under identical conditions, offering a basis for direct comparison.

## Results

### Comparing the ultrastructures and crystallography of modern and fossil *Ammonia*

**Fig. 1:**
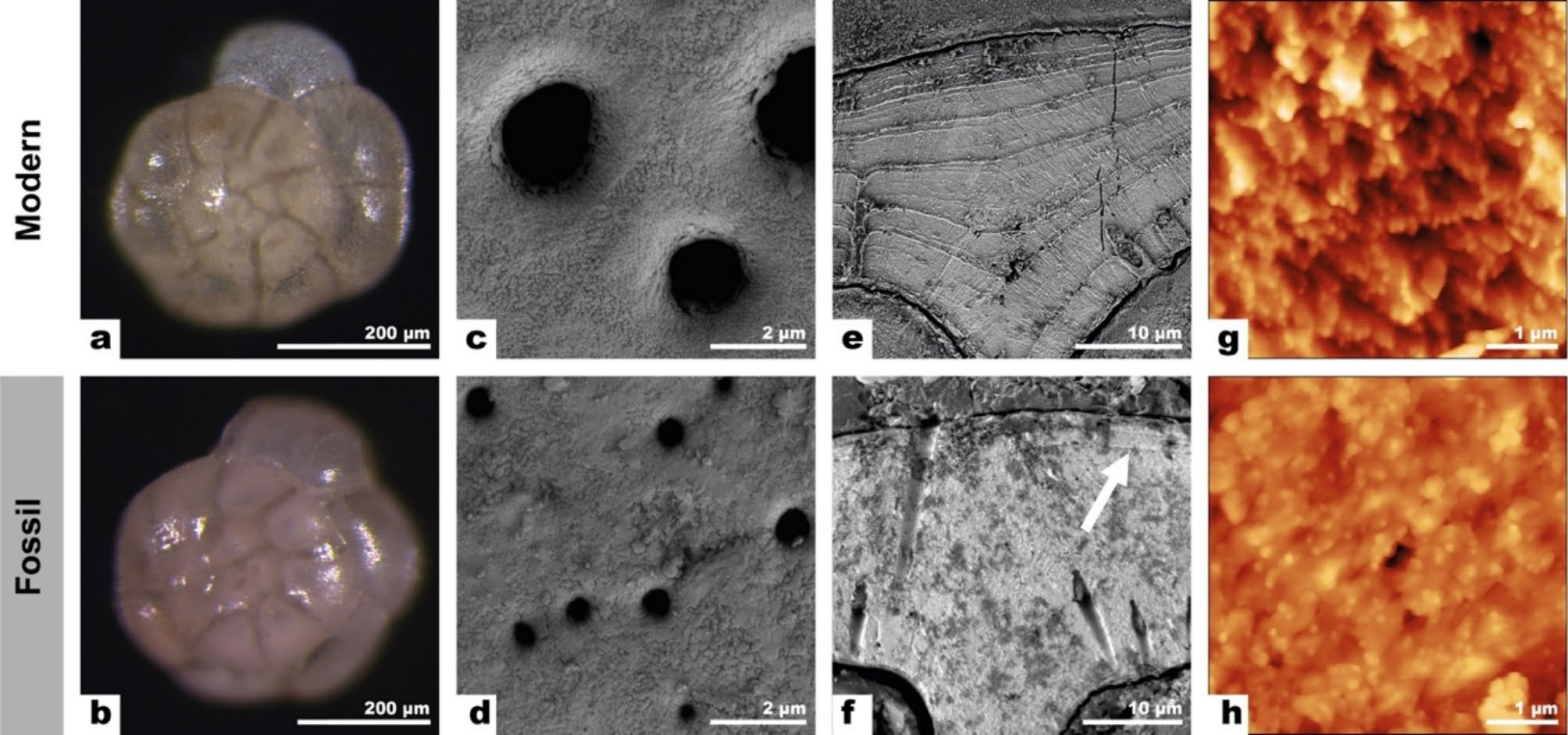
Stereo microscopy, SEM and AFM images comparing microstructures in tests of modern *Ammonia confertitesta* (top row) and fossil *Ammonia beccarii* (bottom row) tests used in the incubation experiments. (a–b) Optical images show that both modern and fossil *Ammonia* appear ‘glassy and pristine’, with pores and chamber divisions clearly outlined. (c–d) High-resolution SEM images of the outer surfaces of modern and fossil tests show comparable surface textures and generally empty pores for both. (e–h) SEM and AFM images of the internal textures of modern and fossil *Ammonia* tests embedded in epoxy, mirror polished and briefly fixed and etched in a solution of glutaraldehyde and acetic acid (see Methods). (e) SEM image of a modern tests showing organic linings are clearly visible as fine ridges. (f) SEM image of a fossil test showing an absence of ridges from organic linings, apart from a few regions indicated with a white arrow. (g and h) AFM imaging of modern and fossil tests show that they have comparable nanogranular textures typical for Rotaliid foraminifera.

A combination of scanning electron microscopy (SEM), atomic force microscopy (AFM), cathodoluminescence (CL) and electron backscatter diffraction (EBSD) imaging were used to compare the ultrastructures of modern *Ammonia confertitesta* and fossil *Ammonia beccarii* used in the incubation experiments, which will henceforth collectively be referred to as *Ammonia.* Optical and SEM images showed that pristine fossil and modern tests appeared highly similar (Fig. 1a–d, Supplementary Figs. 1 and 2).

**Fig. 2:**
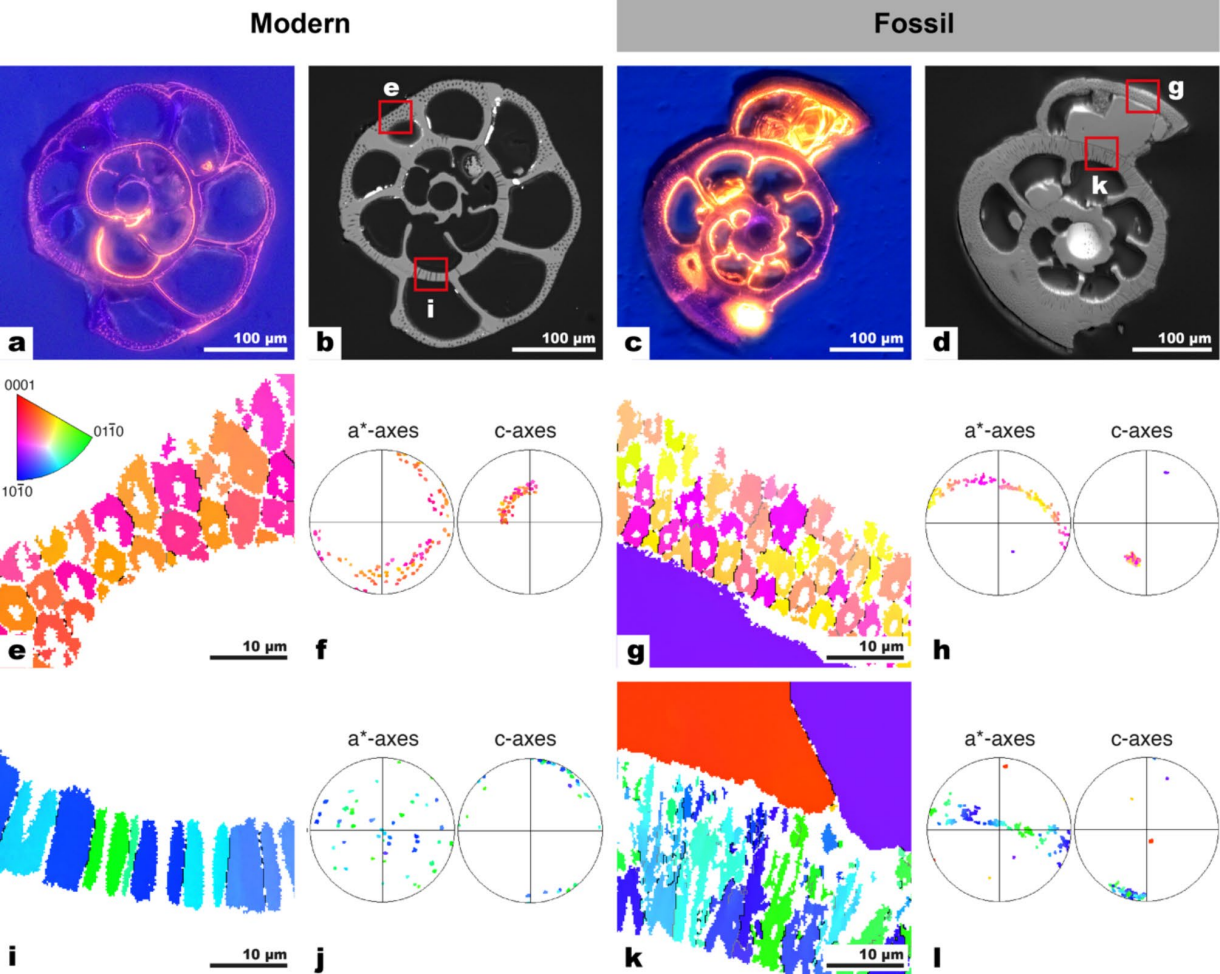
CL and SEM images, along with EBSD maps of polished surfaces comparing modern and fossil *Ammonia*. (a–d) CL and SEM images of modern (a, b) and fossil tests (c, d). (a) The bulk of the modern calcite exhibited blue luminescence apart from orange luminescent bands following the pattern of the organic linings, as well as some orange luminescence around pores. (b) SEM image of the same test as in (a) with the location of subsequent EBSD maps marked with red boxes. (c) The bulk of the fossil test calcite luminesced blue, while the pores, coatings along the insides of tests and the zoned blocky secondary calcite showed bright orange luminescence. (d) SEM image of the same test as in (c) with the with the location of EBSD maps marked with red boxes. (e–l) EBSD maps and pole figures of modern and fossil tests show the cogwheel structures in two section planes: (e–h) the surface perpendicular to the local pore axis, and (i–l) the surface parallel to the local pore axis. Note that while the cogwheel domains were generally smaller in fossil tests, the general shape and orientations of the cogwheel domains were similar to modern tests. EBSD maps of fossil tests (g, k) also showed large blocks of secondary calcite, with different orientations. The EBSD map in (k) has some areas within the test (in white) that could not be indexed.

Both modern and fossil *Ammonia* tests appeared ‘glassy and translucent’^9^ when viewed with an optical microscope (Fig. 1a, b) and high-resolution SEM images showed both had comparable surface textures with minimal overgrowths (Fig. 1c, d). Modern tests had larger pores than fossil tests, but in both cases the pores were empty when viewed from the outside (Fig. 1c, d). In contrast to their pristine outer surfaces, several forms of secondary calcite precipitation were observed on the inner surfaces of fossil tests: as large blocky crystals of secondary calcite (tens of micrometers) partly filling some chambers (see Fig. 2d), as ~ 1 μm euhedral crystals attached to the inside of tests, as thin irregular coatings of calcite, and as a dendritic infilling inside some pores (Supplementary Fig. 3).

To study their internal ultrastructures, modern and fossil tests were embedded in epoxy, mirror polished and treated with a solution of glutaraldehyde and acetic acid that gently etched the test calcite while simultaneously fixing the organic components (cf. Methods). SEM images of the surfaces of modern tests clearly showed a number of organic linings as ridges emerging from the surrounding (etched) calcite (Fig. 1e); the number of linings corresponding to the position of the chamber relative to the last precipitated chamber^20^ (Supplementary Fig. 4). Fossil tests exposed to the same etching and fixation treatment did not show the same organic lining patterns (Fig. 1f). However, between the organic linings high-resolution AFM imaging showed that both modern and fossil tests exhibited the nanocrystalline ultrastructure that is typical of all *Rotaliid* foraminifera (Fig. 1g, h).

Modern tests showed a blue cathodoluminescence with bands of orange that followed the shape of the organic linings and within pores (Fig. 2a). Foraminifera can incorporate soluble Mn^2+^ when it is present in their environment under oxygen-depleted conditions, and this banding could indicate that these foraminifera grew in Mn-rich waters with fluctuating oxygen concentrations^25–27^. The modern tests also showed orange luminescence within pores, indicating some amount of early secondary calcite coating of pore spaces^28^ (Fig. 2a), although SEM images show that pores are not completely infilled (Fig. 1c). The fossil tests luminesced blue and similarly exhibited orange luminescence within pores, with additional bright orange luminescence of the secondary calcite coatings along the inside surfaces of tests and strongly zoned CL patterns within the large blocky pieces of secondary calcite (Fig. 2c).

EBSD was used to examine potential crystallographic changes due to recrystallization between modern and fossil tests. EBSD maps and pole figures were obtained of surfaces both perpendicular and parallel to the local axis of pores in modern and fossil tests (Fig. 2e–l). In these EBSD crystal orientation maps, similar colors indicate regions with similar crystallographic orientations, while different colors indicate adjacent areas with different crystallographic orientations (i.e., where the crystallographic axes misaligned by more than 10°, see Methods). When the imaging surface was perpendicular to the local axis of the pores, the cogwheels formed approximately circular mesocrystals centered around pores in both modern and fossil *Ammonia* (Fig. 2e, g). Mesocrystals are a common feature of biominerals and are defined as superstructures composed of nanocrystals that have a common crystallographic orientation, that are only slightly misaligned with respect to each other. When the surface imaged by ESBD was parallel to the local axis of the pores, the cogwheels appeared as elongated domains parallel to axis of the pores (Fig. 2i, k). For both modern and fossil tests, the c-axis shows a clear preferred orientation perpendicular to the test surface, such that the c-axis rotates with the curvature of the test with the a*-axis randomly oriented on the plane normal to the c-axis (Fig. 2f, h, j, l). The large blocks of secondary calcite infilling in fossil tests (Fig. 2c, d, g, k) had orientations distinct from the adjacent biogenic calcite (Fig. 2h, l).

### NanoSIMS imaging of ^18^O-enrichments in modern and fossil *Ammonia* tests exposed to identical experimental diagenetic conditions

**Fig. 3:**
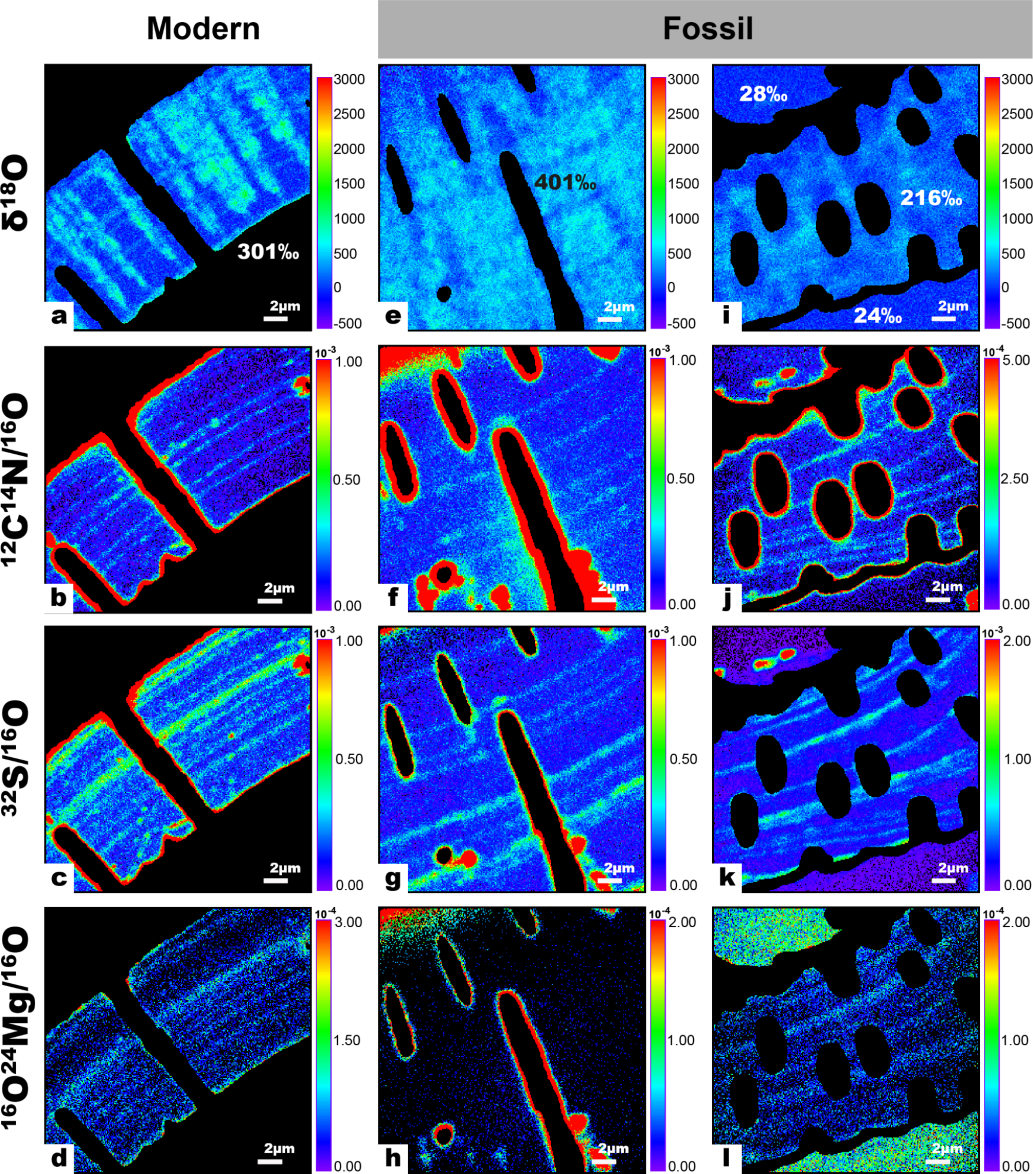
NanoSIMS images of the polished surfaces of modern and fossil *Ammonia* after exposure to artificial seawater (ASW) with a ^18^O/^16^O ratio of 0.30 for 6 days at 90 °C. On figures (a), (e) and (i) the average δ^18^O value of the tests and secondary calcites are reported relative to non-incubated tests and abiotic calcite respectively. (a–d) ^18^O-enrichments and elemental bandings in modern tests. (a) ^18^O-enrichments were heterogenous but occurred predictably along cogwheel structures and organic linings. (e–l) ^18^O-enrichments and elemental bandings in fossil tests. (e) ^18^O-enrichments in fossil tests were also heterogenous but did not show increased ^18^O-enrichment along recognizable ultrastructures. In (e) relatively low ^18^O-enrichments were observed in the areas where the organic linings are normally situated (see CN banding in f). Note the generally low-to-negligible ^18^O-enrichment of the secondary calcite infilling on either side of the test in (i) and the relatively high Mg-content of this secondary calcite (l).

Modern and fossil *Ammonia* tests were exposed to experimental conditions similar to those of a previous study^16^, namely incubation at 90 °C for 6 days in a highly ^18^O-enriched artificial seawater at calcite saturation (^18^O/^16^O = 0.30, Ω_calcite_ = 1). NanoSIMS imaging subsequently permitted the quantitative visualization of the amount of isotopic exchange with the seawater. Because the experiments took place at calcite saturation, no significant changes to test textures due to dissolution or precipitation resulted from these incubations, i.e., the tests remained visually pristine (Supplementary Fig. 5), consistent with observations from similar previous experiments^11,13,16^. NanoSIMS images were obtained of surfaces parallel and perpendicular to the local pore axis (Fig. 3). Consistent with previous results^16^, modern *Ammonia* tests showed spatially pervasive ^18^O-enrichment of the entire test relative to control samples (Fig. 3a). The highest measured ^18^O-enrichments occurred along two main penetration pathways: cogwheel interfaces and organic linings (Fig. 3a), which are common to all rotaliid foraminifera and visualized here with nitrogen measured as ^12^C^14^N^−^ (Fig. 3b). Fossil tests also showed extensive O-isotope exchange throughout the test structure (Fig. 3e, i). However, in contrast to modern tests, the fossil tests had less O-isotope exchange along organic linings (Fig. 3e, i) and no evidence for effective exchange along cogwheel structures (Fig. 3i). Sulfur and magnesium bands (comparable in modern and fossil tests) were not correlated with ^18^O-enrichments (Fig. 3). Secondary, blocky calcite in fossil tests was enriched in Mg relative to the foraminifera tests (Fig. 3l) but did not show detectable ^18^O-enrichment (Fig. 3i).

**Fig. 4:**
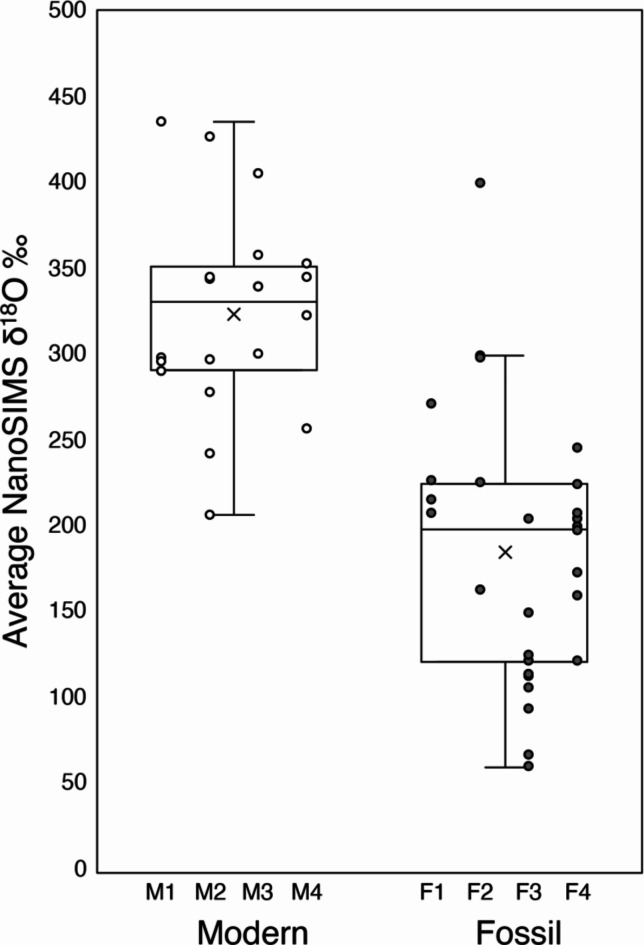
^18^O-enrichments measured with NanoSIMS in modern and fossil tests after exposure to ASW with a ^18^O/^16^O ratio of 0.30 for 6 days at 90 °C. Boxplots show that average ^18^O-enrichment, reported relative to pristine tests of the same species ± 1 standard deviation was 325 ± 58 ‰ (n = 20) for modern *Ammonia* and 186 ± 76 ‰ (n = 28) for fossil *Ammonia*, as indicated by the × symbol. Middle lines = medians, boxes = interquartile ranges, whiskers = minimum and maximum values. The circles indicate individual ^18^O-enrichments for modern (M1 – M4) and fossil (F1 – F4) tests.

The average ^18^O-enrichment, relative to control (i.e., unlabelled) tests of the same species, was 325 ± 58 ‰ (n = 20) for modern *Ammonia* and 186 ± 76 ‰ (n = 28) for fossil *Ammonia* (Fig. 4), which was statistically different (*t* (46) = 6.8, *p* < 0.001). Comparing the average ^18^O-enrichments of individual tests showed that some fossil tests were more reactive than others, with some domains within fossil tests as susceptible to diagenetic isotope exchange as modern tests (Fig. 4). The average δ^18^O of secondary calcite was 11 ± 24 ‰ (n = 10), i.e., effectively zero enrichment within the roughly 30 ‰ (conservative) detection limit of the NanoSIMS operating in this imaging mode.

## Discussion

Biogenic calcites differ from abiotic calcites in their nanogranular textures^21^, distorted crystal lattices^29,30^, heterogenous trace-element chemistry (Fig. 3) and the presence of inter- and intracrystalline organic materials (Fig. 1). Thermodynamics dictates that excess Gibbs free energy associated with these features will drive recrystallisation to an end-product more akin to a homogenous abiotic calcite, which has much lower susceptibility to diagenetic isotope exchange^16^. The question then remains whether fossil biocalcites, which likely have experienced degradation of organic matrices accompanied by ultrastructural changes, are as susceptible to diagenetic isotope exchange as modern biocalcites or if they are more comparable to abiotic calcites.

The multi-analytical imaging approach (SEM, AFM, CL, EBSD, NanoSIMS) employed here demonstrates that the fossil foraminifera tests used in this study were well preserved, though there are some caveats to consider. First, the external textures of tests, which is one of the main criteria for identifying diagenetically altered/unaltered foraminifera to be accepted/rejected for paleo-temperature reconstructions, were highly similar between these fossil and modern tests, both having a glassy appearance (Fig. 1). However, even the glassy fossil tests used here showed clear evidence for secondary calcite precipitation along the insides of tests (Fig. 2d). This would not disqualify these fossil tests from inclusion in paleoclimate reconstruction studies as recently, researchers have been using microspatial in-situ techniques to identify authigenic overprints and select the more pristine parts of tests for isotope analyses^31–33^.

The secondary calcite in this study was highly luminescent and occurred within pores of both modern and fossil tests, as well as large blocky calcite infilling of fossil tests (Fig. 2a, c). However, the fossil test calcite itself was not luminescent, which is a strong indication that recrystallization did not occur^28^. Furthermore, NanoSIMS imaging showed that this blocky secondary calcite has a higher Mg concentration (Fig. 3l) than the test calcite, even including naturally occurring Mg-banding in foraminifera^20^ (Fig. 3d, h, l). Overall, the evidence from optical and SEM images of the outer surfaces of tests, as well as CL and NanoSIMS imaging attest to an excellent preservation state of these fossil tests.

However, SEM images of the internal textures of tests revealed differences between modern and fossil test calcite, primarily related to the degradation of organic matter. Fixation and etching of modern tests revealed prominent ridges at the location of the organic linings (Fig. 1e). Apart from occasional ridges (e.g., white arrow in Fig. 2f), similar regions in fossil tests did not show much evidence for the preservation of organic linings. NanoSIMS maps nevertheless showed that CN-banding was preserved in fossil tests in a manner similar to that of modern tests (Fig. 3). Foraminiferal organic linings are predominantly made up of proteins and polysaccharides^34^ and are known to persist, albeit in increasingly degraded form, for millions of years^35^. However, proteins in fossils invariably degrade into smaller peptides over time^36,37^. In a study of the degradation of intracrystalline proteins in fossil brachiopods, Walton^22^ found that 95% of proteins were degraded into their constituent amino acids after only 120 kyrs. Proteins that are bound to mineral surfaces, such as the charged proteins in organic linings thought to be responsible for directing nucleation of calcite^38^, are among the most resistant to degradation^39^. The lack of relief in etched samples in the SEM and NanoSIMS images suggests that the bulk of the organic linings was degraded, but the organic molecules bound to the test calcite were preserved and are the source of the nitrogen (CN^–^) counts in the NanoSIMS maps (Fig. 3). There are no obvious gaps in the tests where the organic matter would normally be found (Fig. 2f). It is possible that the voids created by the loss of organic matter were either sealed due to pressure or filled by trace amounts of secondary calcite during the natural fossilization process before the start of the experiment, although this is not observable as enhanced cathodoluminescence. Despite the differences in the internal textures observed with SEM, the AFM imaging indicated no difference between the nanocrystallite structures of modern and fossil tests (Fig. 2g, h).

EBSD mapping offered another relevant comparison between modern and fossil tests and is increasingly being used to identify diagenetic overprints in biominerals. In simulated diagenesis experiments on bivalves, gastropods and corals, EBSD was used to track the subtle reorganization of the aragonitic microstructure well before transformation into calcite^40^. Similarly, Casella and colleagues^41^ used EBSD to compare the textures of naturally and experimentally altered calcitic brachiopod shells. The highly similar EBSD maps for modern and fossils tests show that there was a lack of overt recrystallisation due to diagenesis, although the quantification of some EBSD parameters suggests that the fossil calcite tests were somewhere along a continuum between biogenic and abiotic calcite (see Supplementary Information).

Altogether, NanoSIMS, SEM, AFM, CL and EBSD observations suggest that the fossil *Ammonia* tests used in this study are well-preserved specimens, yet close inspection reveals some subtle alteration, mostly related to the degradation of organic matter and infilling with secondary calcite.

Previous work has established that fluids will exploit two organic-rich ultrastructures common to all hyaline rotaliid foraminifera – cogwheel structures and organic linings – to pervasively penetrate and isotopically exchange with foraminiferal test calcite^16^. It was also postulated that beyond the large surface areas created by the nanogranular structure inherent to foraminifera tests, it is partly the inter- and intracrystalline organic matter that facilitates this isotopic exchange. Biogenic calcite has longer and weaker C-O bonds than abiotic calcite due to the anisotropic lattice distortions caused by intracrystalline organic macromolecules^29,30,42,43^. Thus, this organic matter not only creates pathways for fluids to penetrate into the test structure, but also makes the organic matter-associated calcite more reactive. With the gradual degradation of organic matter in biogenic carbonates during fossilization, some authors have proposed that the anisotropic strain induced by organic molecules may relax and the lattice parameters of biogenic calcite will approach that of inorganically precipitated calcite^44^. Additionally, as organic matter degrades, oxidative reactions producing CO_2_ could lower the local pH, which may increase calcite reactivity at these sites^45^. However, even with a rapid degradation of intercrystalline organics^46–48^ and a – presumably slower – decay of intracrystalline organics over time^22,49^, the question remains how fast fossil biocarbonates continue to exchange isotopically with porewaters.

From NanoSIMS images of the experimentally incubated fossil tests, despite the diminished organic matter content, diagenetic fluids can still penetrate into and exchange with fossil calcite (Fig. 3e, i). The observed ^18^O-enrichment was pervasive with areas of preferential O-isotope exchange, but these patchy enrichments bore no relationship to ultrastructural features such as organic linings, cogwheel structures or elemental banding (Fig. 3). In fact, there seemed to have been a lower rate of exchange at the locations of the partially degraded organic linings (Fig. 3e, f). This contrasts with modern tests that exhibited preferential O-isotope exchange along cogwheel structures and organic linings (Fig. 3a). As shown here and in previous work, secondary abiotic calcite is much less susceptible to isotopic exchange than biogenic calcite^16^. Internal secondary calcite precipitation filling the space after degradation of organic linings could therefore explain the lower O-isotope exchange at these sites (Fig. 3e).

Comparing average NanoSIMS ^18^O-enrichments in experimentally incubated modern and fossil tests, the fossil tests were on average about 40% less enriched (Fig. 4). The lack of overt recrystallization as documented with AFM and EBSD (Figs. 1, 2), suggests that the main difference between the modern and fossil tests used in this study was the reduced amount of organic matter in fossil tests. In other words, the organic matter lost was contributing to around half of the reactivity of foraminiferal calcite. Chanda and colleagues^12^ similarly found differences in the reactivities of modern and middle/late Miocene (ca. 10.4 Myr) fossil foraminiferal calcite to ^45^Ca-spiked fluids, with modern tests reacting about ten times faster than fossil tests. These authors found that shallowly buried (younger) fossil tests incorporated more of the ^45^Ca tracer than more deeply buried (older) tests, and proposed that these differences could be due to these tests having different reactive histories, i.e., that some tests have been partially recrystallized^12^. If the preservation state of the fossil specimens in the Chanda et al.^12^ study is comparable to the fossil tests in the present study, we propose that these differences in diagenetic susceptibility could also be due to the progressive degradation of the inter- and intracrystalline organic material in fossil specimens over time.

Following the death of a marine calcifier, diagenetic processes begin to alter the original isotopic and chemical composition of its biocarbonate remains^50^. As shown here and in our previous work^16^, seawater will exploit organic-rich ultrastructures during early diagenesis to rapidly and pervasively penetrate the bulk of the shell calcite. During burial, as temperature increases and/or pore fluid isotope (and chemical) compositions deviate from those of the original seawater, the isotope disequilibrium between these early diagenetic fluids and the buried biominerals increases, leading to a modification of the isotopic composition of the shells^11,13^. As fossilization progresses, the degradation of intercrystalline organics, such as organic linings in foraminifera, creates secondary porosity, which could be infilled with early diagenetic calcite that may partially reduce the susceptibility of fossil calcite to further diagenetic isotope exchange. But clearly, as shown in this work, these fossil biocalcites did not lose their capability to exchange isotopically with surrounding porewaters.

The present study and that of Chanda and colleagues^12^ show that despite age-related reductions in diagenetic susceptibility, fossil calcite remains much more susceptible to diagenetic isotope exchange than abiotic calcites. AFM images in this study revealed no changes in the nanocrystallite structure between modern and fossil foraminifera. The small sizes of the nanocrystallites (20 to 150 nm) that constitute the bulk of the majority of biocalcites^21^ means that grain boundary and lattice diffusion can measurably alter the isotopic composition of the biocalcites used for paleoclimate reconstructions. Previous work has shown that the effects of grain boundary diffusion alone can, on timescales of hundreds of years, bias paleotemperature reconstructions by up to 1 °C and, given enough time, the effects of lattice diffusion can cause under- or overestimates of the true paleotemperature calculated from oxygen isotope compositions^11,13^. The oxygen diffusion coefficients derived from biocalcites are larger than for abiotic minerals, probably as a consequence of embedded organic matter^13^.

In this study, we used a multi-analytical screening approach (SEM, AFM, CL, EBSD, NanoSIMS) to compare the ultrastructure and crystallography of modern and fossil *Ammonia* species. We investigated oxygen isotope exchange with highly controlled incubation experiments on these materials. Despite substantial (but not complete) degradation of organic matter-rich pathways that facilitate fluid penetration in modern tests and offer more reactive sites for O-isotope exchange, fossil tests exhibited about half the ^18^O-enrichment compared with pristine modern foraminifera tests. This demonstrates that, while early diagenesis will proceed along organic-rich structures in the carbonate skeletons of recently deceased organisms, fossilized tests with diminished amounts of organic matter are far from impervious to the effects of long-term diagenesis. The best (and perhaps only) way to remove this bias from paleo-ocean temperature records calculated from oxygen isotope compositions of carbonate fossils, is to better understand the diagenetic susceptibility of the biogenic carbonates most used in paleoclimate studies and to apply the necessary correction factors to existing data.

## Methods

Two species of *Ammonia* were used for the incubation experiments, modern *Ammonia confertitesta* (previously named *Ammonia tepida* T6) and fossil *Ammonia beccarii*. *Ammonia confertitesta* specimens were obtained from recent tidal sediment in the mudflats of the Bay of Bourgneuf, France. The fossils were collected from the Korytnica Clays at Mt. Łysa (located at GPS position: 50°39′50′′ to 50°40′50′′ N and 20°31′20′′ to 20°33′00′′ E), which are part of a fossil rich facies deposited between 14.8–14.6 Myr^23,24^ in the Korytnica Basin^24,36,51^. Fossils from the Korytnica Clays are exceptionally well preserved due to their rapid deposition in water-impermeable clays, and coral, gastropod and fish otolith fossils often maintain their original aragonitic mineralogy as well as their original nanostructural textures^36^. Fossil specimens from the same collection are housed at the Institute of Paleobiology, Polish Academy of Sciences, Warsaw (abbreviation ZPAL).

### Cleaning procedures and incubation experiments

The procedure below is similar to that for our previous work^11,13,16^. Prior to the incubation experiments, the foraminifera tests were ultrasonicated in methanol and subsequently deionized water (MilliQ) before rinsing in technical grade ethanol and drying overnight at 50 °C. We omitted the oxidative cleaning step that removes organic matter as we previously found that this did not make a significant difference to the O-isotope exchange^16^. For the incubation experiments, a dozen modern and fossil tests were placed in separate arc-welded gold capsules filled with ~ 40 µL of artificial seawater (Ω_calcite_ = 1; 0.6 M NaCl, 0.05 M MgCl_2_), which was enriched in ^18^O to a ^18^O/^16^O ratio of about 0.30, and placed in ovens at 90 °C for 6 days. After removing the samples from the gold capsules, the tests were rinsed successively in artificial seawater, distilled and deionized water, and technical grade ethanol. The tests were then dried overnight at 50 °C and subjected to 24 h of vacuum desiccation at room temperature. Previous work has confirmed that the addition or omission of desiccation steps does not change the δ^18^O value of the incubated foraminifera tests^13^.

### Imaging by SEM, AFM, CL, NanoSIMS and EBSD

Uncoated whole and broken tests were imaged using secondary electrons on a Zeiss Gemini 500 SEM operating at an acceleration voltage of 1 kV and a working distance of 5 mm. For the rest of the analyses a flat imaging surface was required. To produce a smooth cross-sectional surface of the tests, dried and vacuum-desiccated tests were embedded in resin (EpoThin2, Struers) and polished with a series of increasingly finer-grained diamond pastes (15 to 0.25 µm). This was followed by a brief (3 min) colloidal silica polish using a Vibromet2 to achieve a mirror finish.

For SEM and AFM imaging of internal test textures mirror polished samples were briefly immersed in 2 vol % glutaraldehyde + 0.1 vol % acetic acid. Samples for SEM imaging were immersed in this solution for 10 s and those destined for AFM imaging were immersed in the solution for 3 s.

AFM images were taken using an Asylum Research Cypher VRS instrument (Oxford Instruments, United Kingdom) with an ARC2 controller. The silicon AFM probes used for non-contact tapping mode were uncoated and had a resonant frequency of 150 kHz and a spring constant of 9 N/m (supplier Oxford Instruments, model AC200TS).

Optical cathodoluminescence (CL) imaging was performed on polished sample surfaces using the electronics and electron gun of OPEA adapted to the vacuum chamber of CTTL, Technosyn 8200 MkII, mounted on an Olympus light microscope BX51 equipped with a static stage and a high-sensitivity Olympus DP74. The OPEA was operated at 15–20 kV and 0.4–0.6 mA with an unfocused cold cathode electron beam under an air atmosphere at 0.2 torr (about 26.6 Pa).

EBSD imaging was conducted using an Oxford Instrument Tescan Mira LMU on samples that were coated with approximately 5 nm of carbon. During measurements the SEM was operated with a beam energy of 20 keV, a working distance of 23 mm, and a tilt angle of 70°. EBSD maps were acquired with a 200 nm step size. Kikuchi patterns were acquired with a Symmetry EBSD detector and indexed with AzTec analysis software (Oxford Instruments). Map processing was done with CHANNEL 5 HKL software. Individual crystal boundaries (black lines) were defined as having a misorientation angle larger than 10° and subgrain boundaries (grey lines) as having a misorientation angle larger than 2°.

For NanoSIMS imaging, the procedures followed Cisneros-Lazaro et al.^16^. The polished samples were coated with ca. 15 nm Au to reduce charging on the sample surface. A 16 keV Cs^+^ primary ion beam with a focused spot size of approximately 120 nm (~ 3.7 pA on the sample surface) was used to visualize the distribution of ^18^O-enrichment and elemental banding within the tests. Ions of ^16^O^-^, ^18^O^-^, ^12^C^14^N^-^, ^31^P^-^, ^32^S^-^, ^16^O^24^ Mg^-^ were simultaneously counted in the multi-collector system in individual electron multiplier detectors with a mass resolving power of ~ 9000 (Cameca definition). An electron gun was used to compensate for the positive charges that build up on the surface. 25 × 25 µm^2^ images were generated as a raster of 256 × 256 pixels with a dwell-time of 5 ms per pixel. The resulting images were processed using L’IMAGE (Dr. Larry Nittler, Carnegie Institution of Washington, USA). The oxygen isotope compositions of the tests were reported as δ^18^O (in parts-per-thousand) relative to tests of the same species that were not incubated with the ASW, i.e., pristine:

1 $$\delta^{18} {\text{O}} = \left\{ {\left[ {(^{18} {\text{O}}/^{16} {\text{O}})_{{{\text{sample}}}} - (^{18} {\text{O}}/^{16} {\text{O}})_{{{\text{standard}}}} } \right]/(^{18} {\text{O}}/^{16} {\text{O}})_{{{\text{standard}}}} } \right\}{ } \times { }1000$$

## Electronic supplementary material

Below is the link to the electronic supplementary material.

Supplementary Material 1.

## Data Availability

All relevant datasets for this research are included within the manuscript or supplementary information files.
