# Supplementary material for: Fossil biocalcite remains open to isotopic exchange with seawater for tens of millions of years
Source: Sci Rep. 2024 Oct 22;14:24933. doi: 10.1038/s41598-024-75588-7 (PMC11496820; doi:10.1038/s41598-024-75588-7)
Supplement: Supplementary file 1 — Supplementary Material 1. [file 41598_2024_75588_MOESM1_ESM.docx]

**Supplementary Information for:**

**Fossil biocalcite remains open to isotopic exchange with seawater for tens of millions of years**

Deyanira Cisneros-Lazaro^1*^, Arthur Adams^1^, Jarosław Stolarski^2^, Sylvain Bernard^3^, Damien Daval^4^, Alain Baronnet^5^, Olivier Grauby^5^, Lukas P. Baumgartner^6^, Torsten Vennemann^7^, Jo Moore^6^, Claudia Baumgartner^6^, Cristina Martin Olmos^1^, Stéphane Escrig^1^ and Anders Meibom^1,6,*^

^1^ Laboratory for Biological Geochemistry, School of Architecture, Civil and Environmental engineering, Ecole Polytechnique Fédérale de Lausanne, Lausanne, 1015, Switzerland

^2^ Institute of Paleobiology, Polish Academy of Sciences, PL-00-818, Warsaw, Poland

^3^ Institut de Minéralogie, de Physique des Matériaux et de Cosmochimie, Muséum National d’Histoire Naturelle, CNRS, Sorbonne Université, 75005, Paris, France

^4^ Institut des Sciences de la Terre, CNRS - Université Grenoble Alpes, 38058, Grenoble, France

^5^ Centre Interdisciplinaire de Nanosciences de Marseille, CNRS - Aix-Marseille Université, 13288, Marseille, France

^6^ Center for Advanced Surface Analysis, Institute of Earth Science, University of Lausanne, CH-1015 Lausanne, Switzerland

^7^ Institute of Earth Surface Dynamics, University of Lausanne, 1015 Lausanne, Switzerland

* [deyanira.cisneroslazaro@epfl.ch](mailto:deyanira.cisneroslazaro@epfl.ch), +41779494386; anders.meibom@epfl.ch

EPFL ENAC IIE LGB, GR C2 524 (GR building), Station 2, CH-1015 Lausanne

**
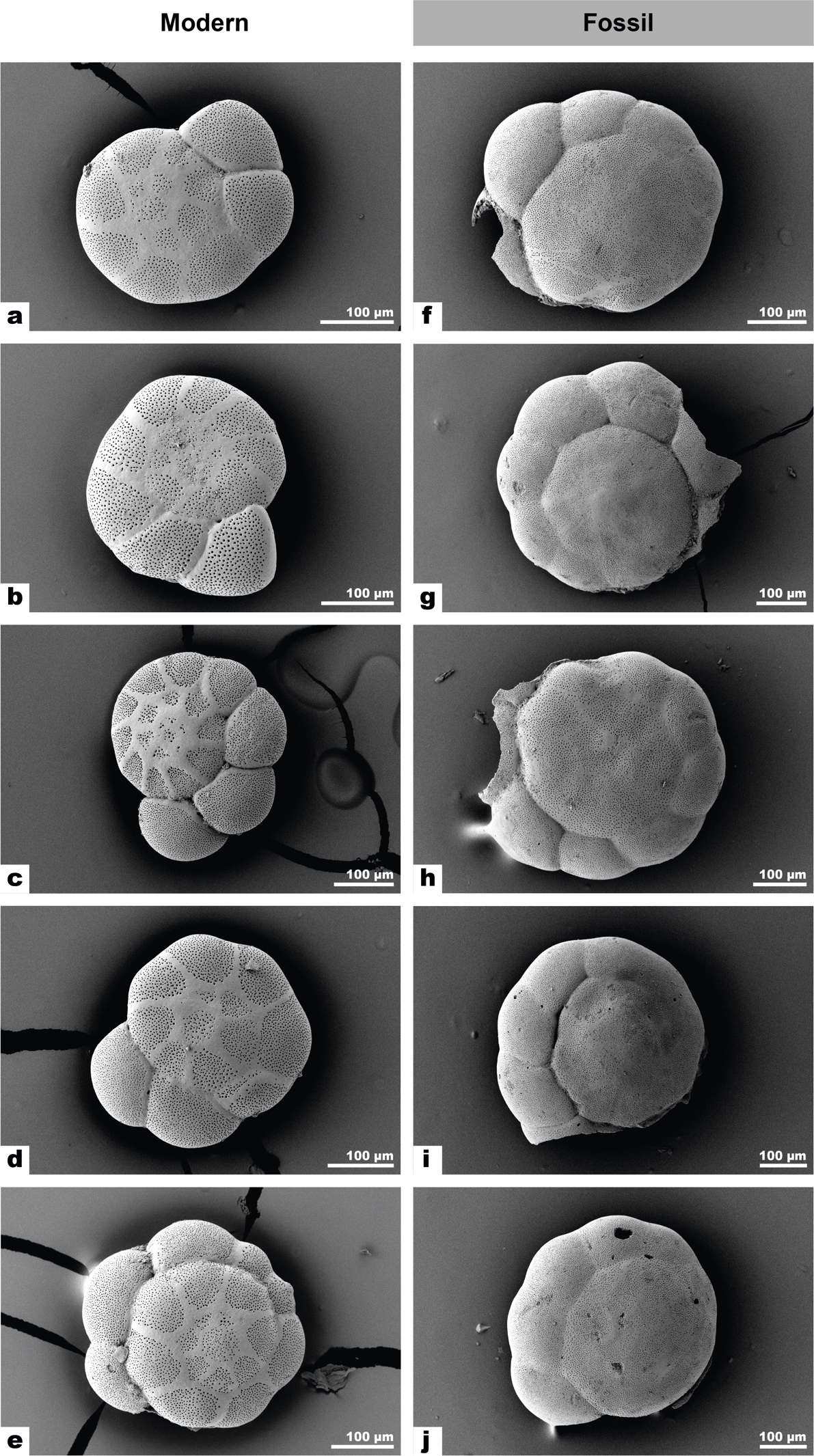
**

**Supplementary Fig. 1** SEM images comparing modern *Ammonia confertitesta* (a–e) and fossil *Ammonia beccarii* (f–j) tests for taxonomic confirmation.

**
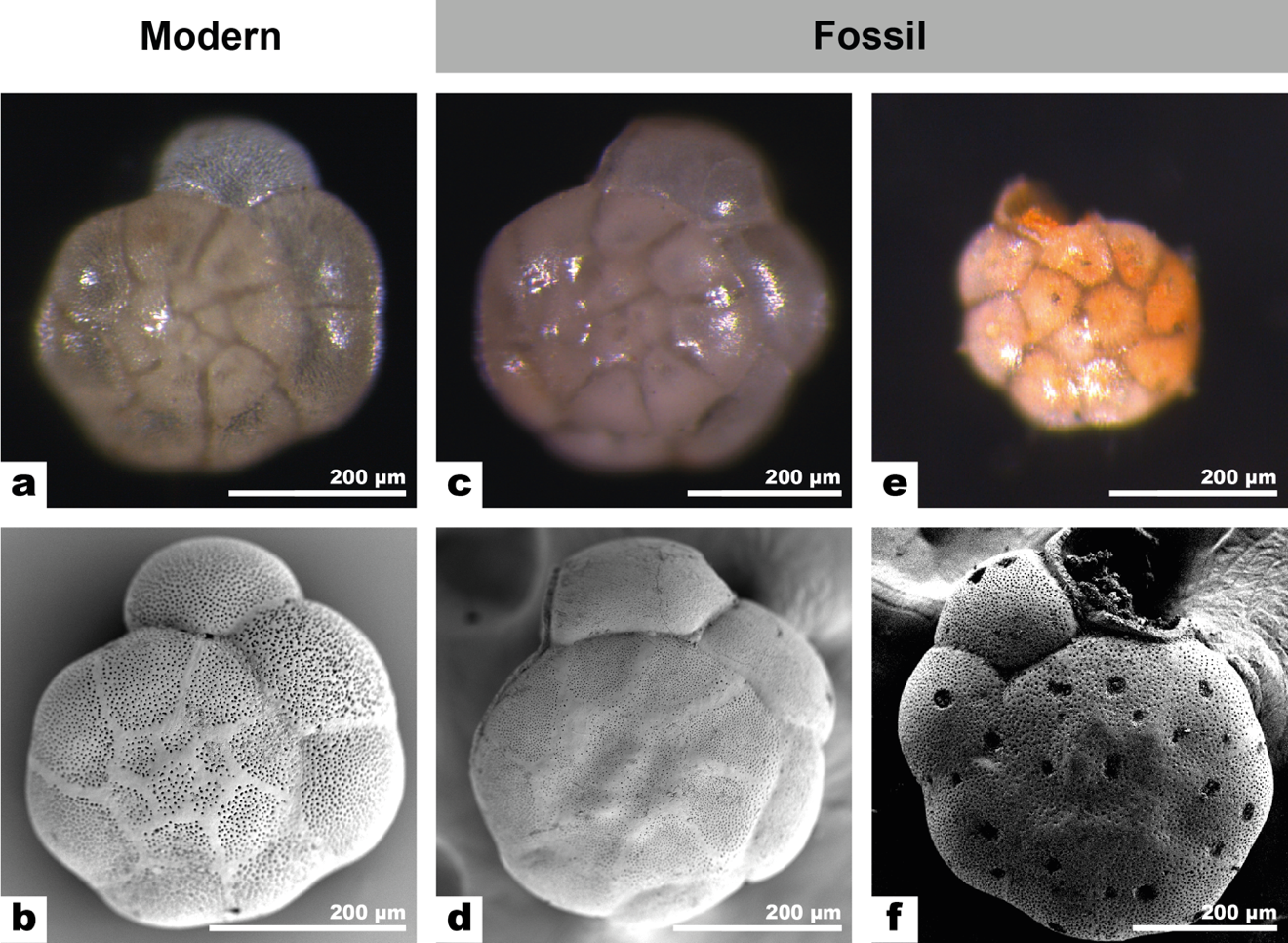
**

**Supplementary Fig. 2** Stereo microscopy and SEM images comparing modern *Ammonia confertitesta* and fossil *Ammonia beccarii* tests. (a–b) A modern test appearing glassy under optical microscopy and showing no egregious overgrowths over the entirety of the test under SEM imaging. (c–d) Only fossil tests that appeared similarly glassy under optical microscopy were selected for the incubation experiments. Most pores were free of infilling and there were only small amounts of overgrowths on the outside surface. (e–f) Fossil tests that appeared ‘frosty’ and showed clear evidence of damage were not selected for the incubation experiments. These tests showed more evidence for pore infilling than more glassy-looking fossil tests.

**
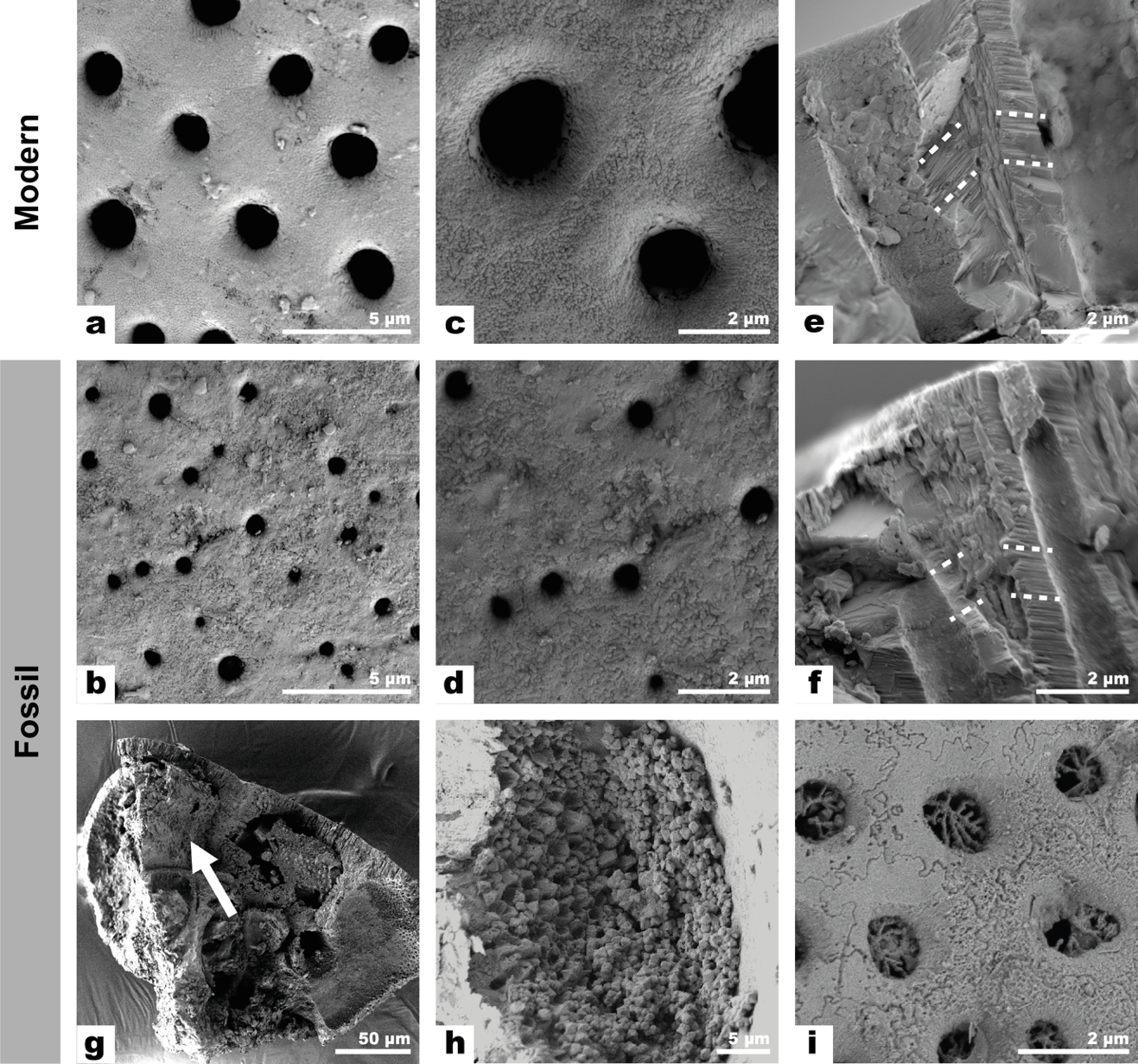
**

**Supplementary Fig. 3** SEM images of the external and internal textures of modern and fossil tests. (a–d) High-resolution SEM images of the outside surfaces showing comparable textures for both modern and fossil tests. (e–f) SEM images of a cross-sectional view of broken chamber walls. Both modern and fossil tests show step-like fractures in opposite directions either side of a vertical cogwheel boundary, as indicated by dashed white lines. (g–i) SEM images of the insides of fossil test chambers. (g) Massive secondary calcite infilling a whole chamber, indicated by the white arrow. (h) Euhedral ca. 1 μm sized secondary calcite grains within the inside of a test. (i) Thin irregular coating of secondary calcite on the inside of a test. A dendritic secondary calcite infilling of the pores was also observed.


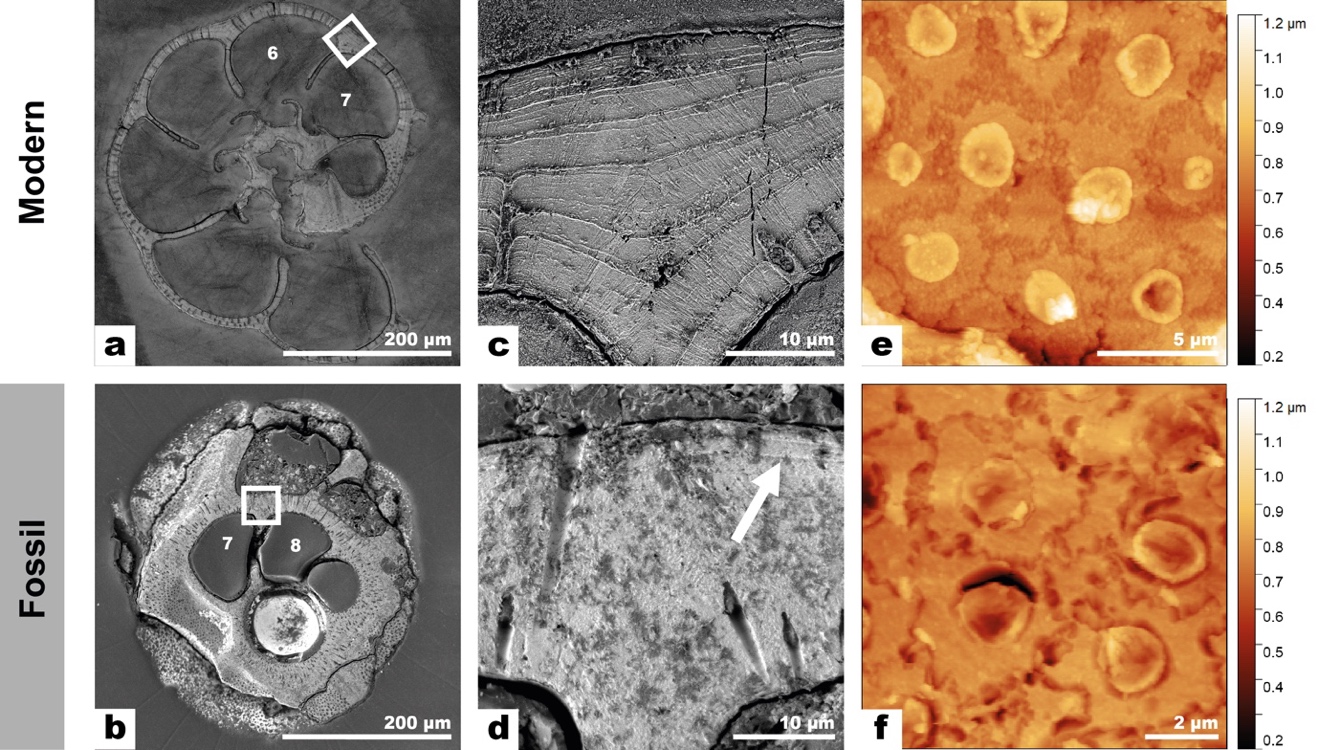


**Supplementary Fig. 4** SEM and AFM images of modern and fossil tests embedded in epoxy, polished, and briefly fixed and etched in a solution of glutaraldehyde and acetic acid. (a–d) SEM images of whole tests showing the number of organic linings expected for the chamber position, with the white squares in (a–b) indicating the location of images c–d. (c–d) Close-up SEM image showing the organic linings clearly standing out as ridges in modern tests, which are not visible in fossil tests apart from at the location of the white arrow. (e–f) AFM images of cogwheel structures in modern and fossil tests. Cogwheel boundaries are present as depression in the surface.

|  | **Porosity %** | **Number of pores** | **Mean pore size (μm^2^)** | **Shapiro-Wilk**  **p-value** | **Kolmogorov-Smirnov**  **p-value** |
| --- | --- | --- | --- | --- | --- |
|  |  |  |  |  |  |
| **Modern** | 12.79 | 18 | 2.11 ± 0.12 | 0.36 | 0.89 |
|  | 18.45 | 24 | 2.04 ± 0.18 | 0.93 | 1.00 |
|  | 9.60 | 12 | 2.40 ± 0.22 | 0.59 | 1.00 |
|  |  |  |  |  |  |
|  | 3.81 | 35 | 0.31 ± 0.02 | 0.00 | 0.42 |
| **Fossil** | 5.94 | 37 | 0.46 ± 0.03 | 0.11 | 0.87 |
|  | 9.56 | 33 | 0.97 ± 0.05 | 0.95 | 1.00 |

**Supplementary Table 1** Porosity and pore size were determined by analysis of SEM images of the penultimate chamber of 3 modern and fossil tests following Petersen et al.^1^ and van Dijk et al.^2^. Shapiro-Wilk and Kolmogorov-Smirnov tests, as well as visual inspection of Q-Q plots were used to test for normality of distribution. Modern tests had larger, more closely spaced pores (i.e., greater porosity) in contrast to fossil tests which had smaller, more sparsely spaced pores (i.e., lower porosity).

**Quantification of EBSD parameters**

Several parameters could be quantified from the EBSD data, which differentiated modern, fossil and secondary calcite. The MUD (multiple of uniform distribution) value, a measure of crystal co-orientation strength, is increasingly used to quantify the degree of diagenetic alteration in biogenic carbonates. A MUD value of 1 indicates a random crystallographic orientation whereas MUD values upwards of 600 are found in inorganically precipitated single crystals of calcite^3^. Whether diagenesis leads to an increase or decrease in the degree of co-orientation seemingly depends on the microstructure, which is a function of the genus. Casella and colleagues^4^ found that MUD values in fossil and hydrothermally altered calcitic brachiopods were lower relative to modern pristine brachiopods. Consistently, Forjanes and colleagues^5^ found that the hydrothermally altered aragonitic bivalve *Artica islandica* had lower MUD values compared to pristine samples. Yet, these authors also found that diagenetic overprints to aragonitic nacre in gastropod *Haliotis ovina* led to a sharp increase in MUD values^5^. In our study, modern *Ammonia* tests had MUD values between 67 and 79 (Supplementary Table 2), which is roughly similar to MUD values of pristine bivalves and gastropods^5^, brachiopods^4^, and foraminifera^6^. Fossil tests had higher MUD values between 132 and 251, with the secondary calcite crystals occurring as blocks of a uniform orientation and showing the highest MUD values between 655 and 694 (Supplementary Table 2), consistent with MUD values of inorganically precipitated calcite^3^.

Differences in MAD values (mean angular deviation, calculated from the EBSD data) between modern and fossil tests and secondary calcite offered another point of comparison. Fossil tests had MAD values of ca. 0.6, closer to secondary calcite (~0.4) than to modern tests values (~1, Supplementary Table 2). This could potentially be explained by the degradation of organic matter and potential infilling of these gaps with secondary calcite, which would decrease the MAD value compared to modern tests. If there was secondary calcite infilling of the gaps created by the loss of organic matter, it either grew epitaxially on the test calcite or was too small to be distinguished by the electron beam in EBSD maps (surface spot-size of 200 nm, but with a larger interaction volume due to the high voltage used). Both MUD and MAD indicate that the fossil calcite tests were somewhere along a continuum between biogenic and abiotic calcite.

In summary, the geometry of cogwheel structures was similar between fossil and modern tests but quantification of EBSD parameters, such as MUD and MAD, indicates that fossil tests have some similarity with inorganically precipitated calcite.

|  | **Viewing plane relative to pores** | | **Number of grains** | **Mean cogwheel size (μm^2^)** | **Max length (μm)** | **S-W**  **p-value** | **K-S**  **p-value** | **Mean MAD** | **SC mean MAD** |
| --- | --- | --- | --- | --- | --- | --- | --- | --- | --- |
|  |  |  |  |  |  |  |  |  |  |
| **Modern** | Perpendicular | | 61 | 12.55 ± 1.31 |  | <0.01 | 0.27 | 1.10 |  |
|  | Parallel | 22 | |  | 14.10 ± 0.79 | 0.05 | 0.07 | 0.98 |  |
|  |  | |  |  |  |  |  |  |  |
| **Fossil** | Perpendicular | | 38 | 11.67 ± 1.44 |  | <0.01 | 0.21 | 0.66 | 0.46 |
|  | Parallel | | 92 |  | 23.35 ± 0.39 | <0.01 | <0.01 | 0.61 | 0.40 |

**Supplementary Table 2** Cogwheel sizes calculated from EBSD data. Reported as mean ± standard error (SE). S-W: Shapiro-Wilk normality test. K-S: Kolmogorov–Smirnov normality test. MAD: Mean angular deviation. SC: Secondary calcite. Bold values indicate a non-normal distribution.

**
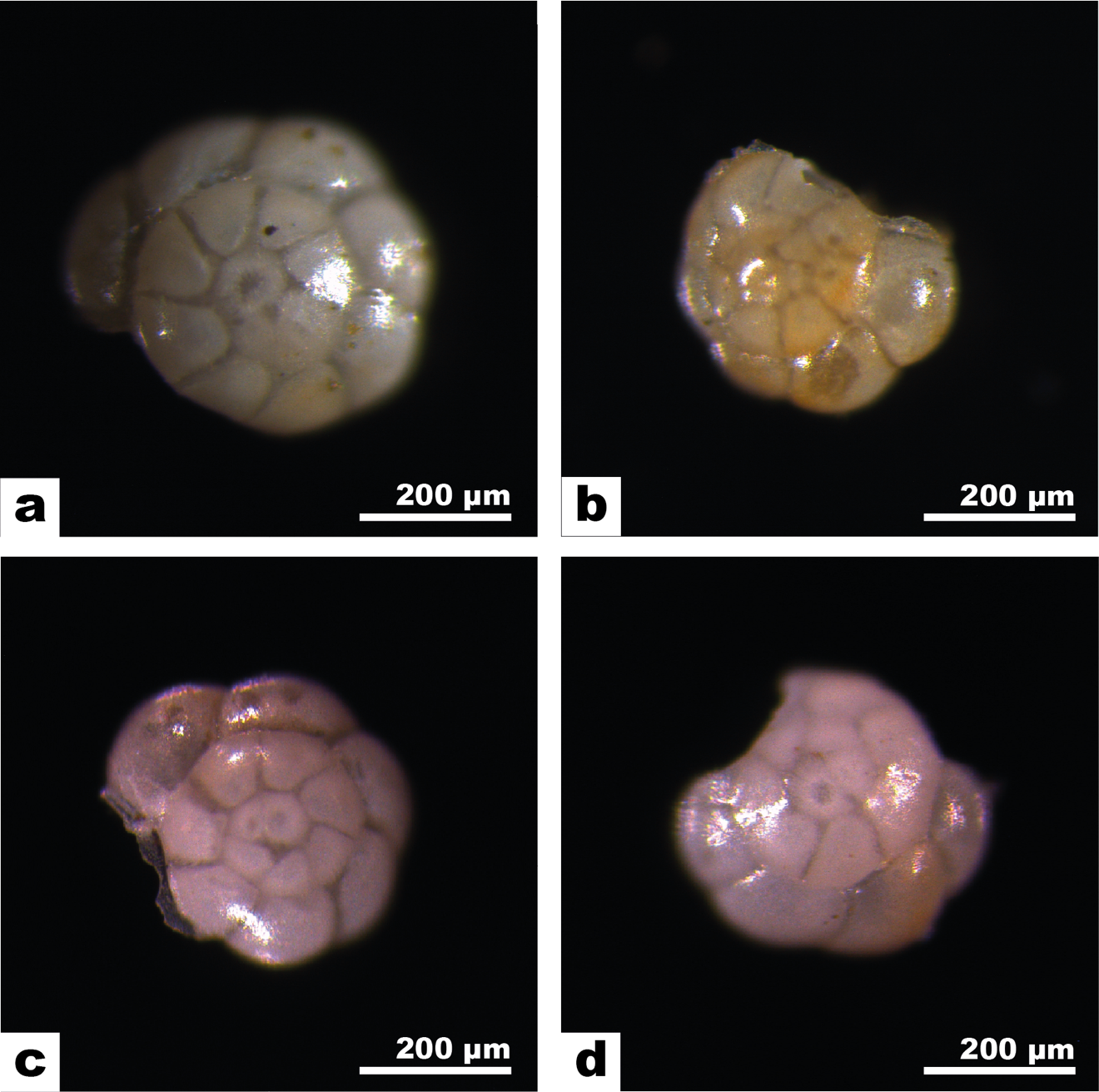
 Supplementary Fig. 5** ﻿Stereo microscopy images of two *Ammonia beccarii* tests before (top row) and after (bottom row) exposure to artificial seawater with a ^18^O/^16^O ratio of 0.30 for 6 days at 90 °C. Note that after the incubation experiment the tests were more fragile and younger chambers were often lost. All images were taken under comparable imaging conditions with the same camera and at the same scale.

|  | **Average NanoSIMS δ^18^O (‰)** | | | |  |
| --- | --- | --- | --- | --- | --- |
| **Species** | *Ammonia confertitesta* | | *Ammonia beccarii* | | *Secondary calcite in Ammonia beccarii* |
|  | M1 | 299 | F1 | 227 | 28 |
|  | M1 | 437 | F1 | 208 | 24 |
|  | M1 | 296 | F1 | 272 | 6 |
|  | M1 | 291 | F1 | 216 | 16 |
|  | M2 | 429 | F2 | 401 | -12 |
|  | M2 | 243 | F2 | 300 | 3 |
|  | M2 | 345 | F2 | 299 | -20 |
|  | M2 | 279 | F2 | 226 | 39 |
|  | M2 | 207 | F2 | 163 | 44 |
|  | M2 | 346 | F3 | 122 | -29 |
|  | M2 | 297 | F3 | 204 |  |
|  | M3 | 359 | F3 | 125 |  |
|  | M3 | 340 | F3 | 60 |  |
|  | M3 | 340 | F3 | 106 |  |
|  | M3 | 406 | F3 | 150 |  |
|  | M3 | 301 | F3 | 67 |  |
|  | M4 | 257 | F3 | 113 |  |
|  | M4 | 346 | F3 | 114 |  |
|  | M4 | 354 | F3 | 94 |  |
|  | M4 | 323 | F4 | 246 |  |
|  |  |  | F4 | 173 |  |
|  |  |  | F4 | 122 |  |
|  |  |  | F4 | 225 |  |
|  |  |  | F4 | 200 |  |
|  |  |  | F4 | 204 |  |
|  |  |  | F4 | 208 |  |
|  |  |  | F4 | 198 |  |
|  |  |  | F4 | 160 |  |
|  |  |  |  |  |  |
| **Average** |  | 325 |  | 186 | 11 |
| **SD** |  | 58 |  | 76 | 24 |
| **n** |  | 20 |  | 28 | 10 |

**Supplementary Table 3** Summary table of the oxygen isotope measurements and average δ^18^O of modern and fossil *Ammonia* tests as well as secondary calcite precipitations (associated with fossil tests) incubated for 6 days at 90 °C in artificial seawater with a 18O/16O ratio of 0.30. The NanoSIMS measurements each reflect the δ^18^O measured over a 25 by 25 um^2^ area. M# and F# refer to individual modern and fossil tests respectively.

**SI References**

1. Petersen, J. *et al.* Improved methodology for measuring pore patterns in the benthic foraminiferal genus Ammonia. *Mar. Micropaleontol.* **128**, 1–13 (2016).

2. van Dijk, I., Raitzsch, M., Brummer, G.-J. A. & Bijma, J. Novel Method to Image and Quantify Cogwheel Structures in Foraminiferal Shells. *Front. Ecol. Evol.* **8**, 1–13 (2020).

3. Yin, X. *et al.* In fluence of Gelatin−Agarose Composites and Mg on Hydrogel-Carbonate Aggregate Formation and Architecture ́. *Cryst. Growth Des.* **19**, 5696–5715 (2019).

4. Casella, L. A. *et al.* Micro- and nanostructures reflect the degree of diagenetic alteration in modern and fossil brachiopod shell calcite : A multi-analytical screening approach ( CL , FE-SEM , AFM , EBSD ). *Palaeogr. Palaeoclimatol. Palaeoecol.* **502**, 13–30 (2018).

5. Forjanes, P., Roda, M. S., Greiner, M., Griesshaber, E. & Lagos, N. A. Experimental burial diagenesis of aragonitic biocarbonates : from organic matter loss to abiogenic calcite formation. *Biogeosciences* **19**, 3791–3823 (2022).

6. Yin, X. *et al.* Calcite crystal orientation patterns in the bilayers of laminated shells of benthic rotaliid foraminifera. *J. Struct. Biol.* **213**, (2021).
